# Co-Conserved Features Associated with *cis* Regulation of ErbB Tyrosine Kinases

**DOI:** 10.1371/journal.pone.0014310

**Published:** 2010-12-13

**Authors:** Amar Mirza, Morad Mustafa, Eric Talevich, Natarajan Kannan

**Affiliations:** 1 Department of Biochemistry and Molecular Biology, University of Georgia, Athens, Georgia, United States of America; 2 Institute of Bioinformatics, University of Georgia, Athens, Georgia, United States of America; University of Oxford, United Kingdom

## Abstract

**Background:**

The epidermal growth factor receptor kinases, or ErbB kinases, belong to a large sub-group of receptor tyrosine kinases (RTKs), which share a conserved catalytic core. The catalytic core of ErbB kinases have functionally diverged from other RTKs in that they are activated by a unique allosteric mechanism that involves specific interactions between the kinase core and the flanking Juxtamembrane (JM) and COOH-terminal tail (C-terminal tail). Although extensive studies on ErbB and related tyrosine kinases have provided important insights into the structural basis for ErbB kinase functional divergence, the sequence features that contribute to the unique regulation of ErbB kinases have not been systematically explored.

**Methodology/Principal Findings:**

In this study, we use a Bayesian approach to identify the selective sequence constraints that most distinguish ErbB kinases from other receptor tyrosine kinases. We find that strong ErbB kinase-specific constraints are imposed on residues that tether the JM and C-terminal tail to key functional regions of the kinase core. A conserved RIxKExE motif in the JM-kinase linker region and a glutamine in the inter-lobe linker are identified as two of the most distinguishing features of the ErbB family. While the RIxKExE motif tethers the C-terminal tail to the N-lobe of the kinase domain, the glutamine tethers the C-terminal tail to hinge regions critical for inter-lobe movement. Comparison of the active and inactive crystal structures of ErbB kinases indicates that the identified residues are conformationally malleable and can potentially contribute to the *cis* regulation of the kinase core by the JM and C-terminal tail. ErbB3, and EGFR orthologs in sponges and parasitic worms, diverge from some of the canonical ErbB features, providing insights into sub-family and lineage-specific functional specialization.

**Conclusion/Significance:**

Our analysis pinpoints key residues for mutational analysis, and provides new clues to cancer mutations that alter the canonical modes of ErbB kinase regulation.

## Introduction

The epidermal growth factor receptor (EGFR) and related kinases, ErbB2, ErbB3, and ErbB4 (collectively called the ErbB family) [1], are key components of our cellular machinery that control signaling pathways associated with cell migration, proliferation, and differentiation. Understanding how ErbB kinases respond to specific activation and regulatory signals in these pathways is essential for the development of new therapies for human cancers that are associated with abnormal regulation of ErbB kinase activity [2]. The domain architecture of ErbB kinases, like most receptor tyrosine kinases (RTKs), is characterized by an extracellular ligand-binding domain, a transmembrane domain, a juxtamembrane (JM) segment, a kinase domain, and a COOH-terminal tail (C-terminal tail). The kinase domain adopts a bi-lobal structure consisting of an N-terminal ATP-binding lobe (N-lobe) and a C-terminal substrate-binding lobe (C-lobe) [3], [4].

Extensive studies on the extracellular ligand-binding domain [5], [6], [7], [8], [9] and more recently on the intracellular kinase domain of EGFR [10], [11], [12] have provided key insights into how EGFR converts an extracellular signal into an intracellular response. Ligand binding to the extracellular receptor induces dimerization and activation of the intracellular kinase domain, which, upon activation, autophosphorylates conserved tyrosine residues in the C-terminal tail [13], [14]. Autophosphorylation of the tyrosine residues activates downstream signaling pathways by recruiting signaling and docking proteins to the C-terminal tail [2], [15], [10]. In the absence of an extracellular ligand, EGFR is maintained in an inactive dimeric form [11], which prevents formation of the active dimer. ErbB3 is believed to diverge from this canonical mechanism of action because of its inability to catalyze phosphoryl transfer [16]. Recent studies, however, have challenged this view by showing low, but detectable levels of ErbB3 autophosphorylation [17].

A key step in ErbB kinase signaling is the activation of the intracellular kinase domain, which is achieved by an intermolecular interaction between two kinase molecules in an asymmetric dimer [10]. In the asymmetric dimer, the C-lobe of one kinase molecule (the “activator”) allosterically activates the other (“receiver”) by inducing conformational changes in key regions of the receiver [10]. In particular, the regulatory C-helix in the N-lobe of the receiver kinase switches from an inactive “out” to an active “in” conformation upon dimerization, and the flexible activation loop in the C-lobe of the kinase switches from a substrate-inaccessible conformation to a substrate-accessible conformation [10], [11], [12], [18]. Also, the N-lobe of the kinase domain moves from an ATP-accessible “open” conformation to an ATP-inaccessible “closed” conformation [15], [19], [20]. These conformational changes, which occur upon activation of many protein kinases [21], [22], [23], [24], are tightly regulated to avoid physiological catastrophes [25], [26].

The catalytic activity of EGFR is also regulated by conformational changes in the JM and C-terminal tail—two sequence segments flanking the kinase domain. The JM segment functions as an activation domain [12] by facilitating the formation of the asymmetric dimer [11]. Specifically, the JM segment of the receiver docks to the C-lobe of the activator to stabilize the asymmetric dimer [11], [12]. This docking interaction is prevented in the inactive dimer of EGFR [11], where the JM docking surface on the C-lobe is shielded by the C-terminal tail. Presumably, the conformational changes associated with the JM and C-terminal tail during kinase activation are closely coupled with the conformational changes in the kinase core (described above) for the tight regulation of kinase activity [27], [28]. The atomic details of how this coupling is achieved are not fully understood.

Receptor tyrosine kinases (RTKs) outside of the ErbB family also contain flexible JM and C-terminal tail segments that play important regulatory roles. In c-KIT and Ephrin receptor tyrosine kinases, for example, the JM segment plays an autoinhibitory role, in contrast to its activating role in EGFR, by interacting with the active site [29] and the substrate-binding regions of the kinase domain [30]. Likewise, the C-terminal tail in Tie2 inhibits catalytic activity by an autoinhibitory mechanism [31], which is distinct from EGFR [27]. Thus, individual RTKs have evolved unique mechanisms to regulate catalytic activity by the JM and C-terminal tails. Information regarding these family-specific regulatory mechanisms are encoded in the protein sequences—the cell's own medium for specifying molecular mechanisms. However, despite the availability of RTK sequences from diverse organisms (∼3000 sequences), the sequence features that contribute to the unique modes of regulation in individual RTKs have not been systematically delineated.

We have shown using several case studies that Bayesian analysis of the evolutionary constraints distinguishing functionally divergent kinases is a viable approach for investigating the functional specificity of kinases in signaling pathways [32], [33]. Using this approach, we recently identified that a conserved C-terminal tail which wraps around the kinase core of AGC kinases is a distinguishing feature of the AGC group [34]. This study also revealed novel AGC kinase-specific motifs in the C-terminal tail that were experimentally shown to be important for AGC kinase functions [35], [36]. In this study, we compare the functional constraints acting on ErbB and related RTKs to identify the key residues/motifs that contribute to ErbB kinase functional divergence. We show, for the first time, that nearly all the residues that distinguish ErbB kinases from other RTKs are involved in tethering the JM and C-terminal tail to key functional regions of the kinase core. Analysis of these tethering interactions in light of the wealth of structural and functional data available on the ErbB kinases suggests a model in which the identified residues contribute to ErbB kinase functional specialization by facilitating a unique *cis* interaction between the kinase core and the flanking JM and C-terminal tails. Our analysis provides new testable hypotheses regarding the *cis* regulation of the kinase core by the JM and C-terminal tails, and provides new insights into cancer mutations that alter this mode of regulation.

## Results and Discussion

### A co-conserved sequence pattern characteristic of the ErbB kinase domain

**Figure 1:**
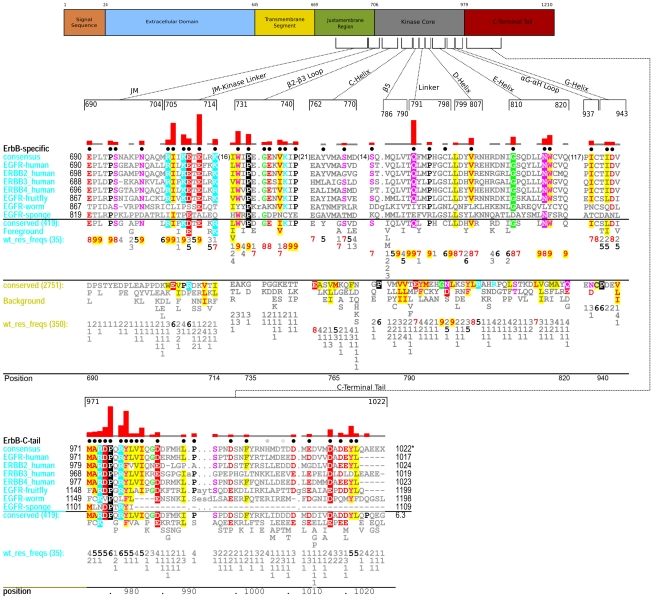
Contrast Hierarchical Alignment showing sequence patterns that most distinguish ErbB kinases (foreground alignment) from other receptor tyrosine kinase (RTK) sequences (background). The residues identified by the Bayesian pattern partitioning procedure (see Methods) as distinctive of the ErbB family are indicated by black dots above the alignment. The histograms on top of the alignment indicate the degree to which residue composition in the foreground alignment (ErbB sequences) contrast with residues observed at the corresponding position in the background alignment (other RTKs). The foreground set includes the sequences shown in the alignment and others whose conservation levels are denoted by the consensus pattern and corresponding weighted residue frequencies (wt_res_freqs) below the alignment. Residue frequencies are indicated in integer tenths where, for example, a ‘6’ indicates that the corresponding residue directly above it occurs 60-70% of the weighted sequences. The number of weighted sequences and the total number of alignment sequences are indicated in parentheses next to ‘wt_res_freqs’ and ‘conserved’, respectively. The background alignment and the corresponding residue frequencies are shown directly below the foreground alignment. The structural location of the ErbB kinase-conserved residues and the overall domain organization of the ErbB family are shown above the alignment. The numbering used in the alignment and in the text is according to the pre-mature EGFR numbering, which includes the 24 amino acid signaling sequence. A background alignment for the C-terminal tail region is not shown because the C-terminal tail of ErbB kinases shares no detectable sequence similarity with the C-terminal tail of other RTKs. Thus, a standard background alignment consisting of protein sequences from NCBI-nr database was used to quantify the constraints acting on the C-terminal tail residues. The NCBI sequence identifiers used in the query display alignment are: EGFR-human: 134104655; ERBB2_human: 119533; ERBB3_human: 119534; ERBB4_human: 3913590; EGFR-fruitfly: 4588511; EGFR-worm: 212645651; EGFR-sponge: 18146642.

To identify which sequence features most distinguish ErbB kinases from other RTKs, we measured and analyzed the selective constraints imposed on ErbB kinase sequences from diverse organisms (see Methods). These constraints generally correspond to residues that are highly conserved within the ErbB family, but strikingly different in RTKs outside of the ErbB family (Figure 1). Within the catalytic core, these residues correspond to W731, P733, G735, E736, V738, K739, P741 in the β2-β3 loop; Y764 and S768 in the C-helix; S784 in the β4-β5 loop; Q791, P794, C797 in the inter-lobe linker; V802 in the D-helix; G810, N816,W817 in the E-helix; P848 in the β8 strand (not shown); L861 in the activation loop (not shown); I/V904 in the F-helix (not shown); I938 in the αG-αH loop; I/L941 and D942 in the H-helix. Among these residues, Q791 in the inter-lobe linker contributes the most (indicated by the height of the histogram in Figure 1) to ErbB functional divergence, since none of the RTKs outside of the ErbB family conserve a glutamine at the 791 position (Background alignment in Figure 1). The residues described above also distinguish ErbB kinases from non-receptor tyrosine kinase (NRTK) sequences, as NRTKs also conserve strikingly different residues at these positions. The only exceptions are W731 in the β2-β3 loop and L861 in the activation loop. These two residues are conserved in ErbB's as well as in some NRTKs.

### The JM and C-terminal tail contribute to ErbB kinase functional divergence

In addition to the kinase domain, strong ErbB-specific constraints are also imposed on residues flanking the kinase core, namely, the Juxtamembrane segment, the JM-kinase linker and the COOH-terminal tail.

#### ErbB-specific constraints in the JM and JM-kinase linker

The JM segment is conserved across diverse organisms within the ErbB family (Figure 1). However, across RTKs, the JM segment displays little or no detectable sequence similarity. This indicates that the JM segment is unique to the ErbB family and likely contributes to its functional divergence. Some of the most distinguishing residues in the JM region include: E690, P694, S695 and N700. Unlike the JM, the JM-kinase linker of ErbB kinases share significant structural similarity with the JM-kinase linker of other RTKs [37], despite very low sequence similarity. This is indicated by the shared hydrophobic residues (L/I 707, L712) between ErbB kinases (Foreground alignment in Figure 1) and RTKs (Background alignment in Figure 1). The JM-linker region also contains several residues that distinguish ErbB kinases (Foreground) from other RTKs (Background). These include: R705, I706, K708, E709, E711 and K714 (Figure 1).

#### The C-terminal tail is a distinguishing feature of ErbB kinases

The C-terminal tail is also a distinctive feature of the ErbB family. In particular, the sequence segment immediately following the kinase domain (residues 971-1020 in Fig 1) is highly conserved in ErbB kinases, but strikingly different in RTKs outside of the ErbB family. The C-terminal tail segment is also co-conserved with key regions of the kinase domain (see below). Some of the distinctive residues/motifs in the C-terminal tail segment include: [MF][AC][RK]DPxR[YF]LVI motif in the beginning of the C-tail, D/E994 and F/L997 in the middle, and [DE]x[DE]xYL motif at the C-terminal end (Figure 1).

### Lineage and sub-family-specific variations within the ErbB family

**Figure 2:**
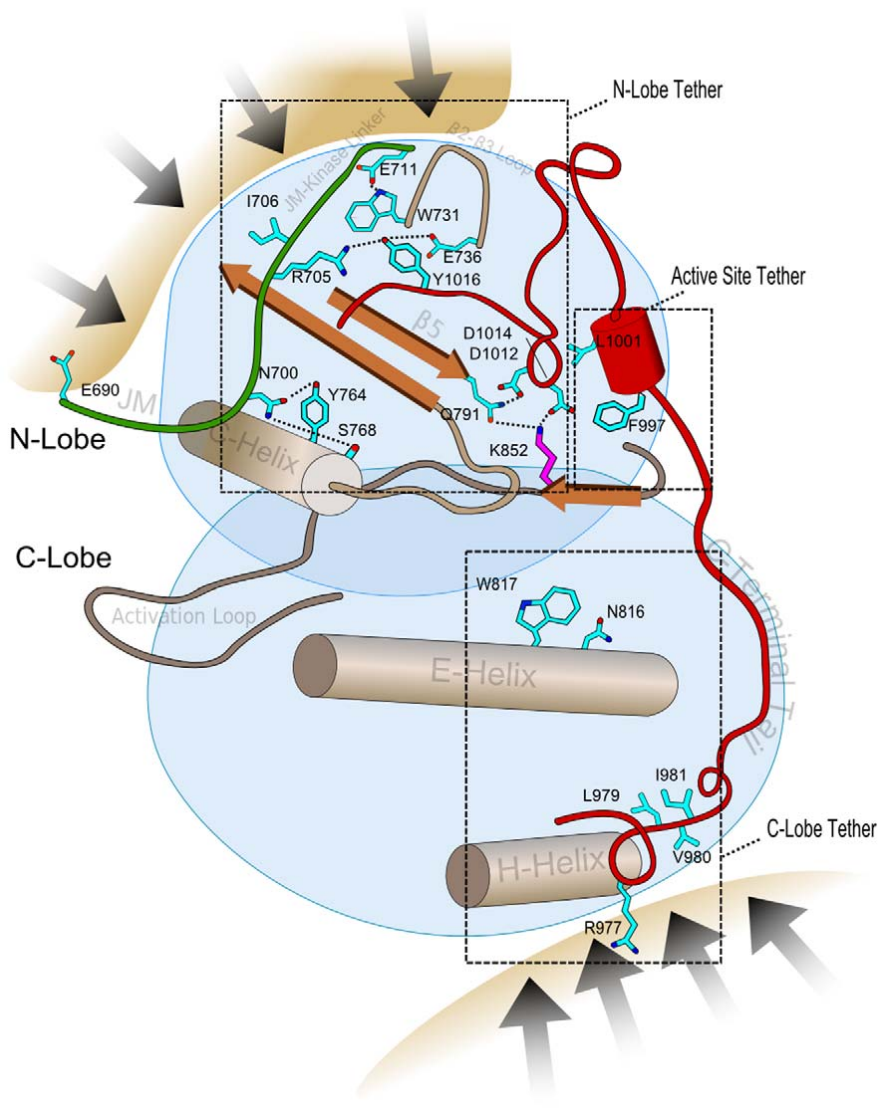
A schematic of the ErbB kinase domain showing the distinctive ErbB residues and associated interactions (based on PDB: 2J5F). These residues are broadly classified into three categories based on their structural location and their role in tethering the JM and C-terminal tail: (i) N-lobe tether (NLT), (ii) Active site tether (AST) and (iii) C-lobe tether (CLT). The residues are shown in stick representation. ErbB kinase-conserved residues are colored in cyan and kinase conserved residues are colored in magenta. The sites of homo/hetero-dimerization are shown by dark arrows.

The ErbB prototypic features, described above, are generally well conserved across diverse eukaryotic phyla (Figure 1). However, some lower eukaryotes and parasitic worms diverge from the canonical ErbB features, and display correlated sequence changes in the JM, kinase, and C-terminal tail regions (Figure 1). For example, a distinctive glutamine (Q791) in the inter-lobe linker region is conserved as a glutamate (E) in sponges (Figure 1). This change is correlated with the absence of the C-terminal tail [DE]x[DE]xYL motif (Figure 1), which typically interacts with Q791 in mammalian ErbB's (Figure 2). Thus, the correlated sequence change observed in the kinase and C-terminal tail suggests possible co-evolution of these two regions during mammalian ErbB kinase evolution.

ErbB3, an atypical member of the ErbB family, also displays significant variations in some of the canonical ErbB motifs/residues. For example, a phosphorylatable tyrosine (Y1016) within the C-terminal tail [DE]x[DE]xYL motif is conserved as an aspartate (D) in ErbB3. Likewise, ErbB3 and ErbB4 replace a canonical tyrosine (Y764) in the C-helix by a leucine (L) (Figure 1). The structural and functional implications of these family-specific variations are discussed in the sections below.

### ErbB conserved residues are frequently mutated in human cancers

Since ErbB kinases are one of the most frequently mutated gene families in human cancers, we investigated whether any of the identified ErbB conserved residues are among those known to be associated with human cancers. Mapping of somatic mutations identified in the ErbB family (see Methods) to available crystal structures indicates that several of the ErbB kinase conserved residues are indeed mutated in human cancers. S768 in the C-helix, and L861 in the activation loop, are two of the most frequently mutated residues in EGFR (Table 1). In addition, ErbB conserved residues in the JM-kinase linker and β2-β3 loop are also frequently mutated in lung, esophagus and upper digestive track cancers (Table 1). The structural/functional impacts of these mutations, however, are not fully understood.

**Table 1:** Somatic mutations in EGFR targeting the canonical ErbB kinase-specific residues.

Mutation	Cancer Primary Site	Structural location
**P694L(1)** **P694S(1)**	Lung	JM
**S695G(1)**	Thyroid	JM
**I706T(1)**	Lung	JM-kinase linker
**K708M(1)**	Lung	JM-kinase linker
**E709A(8)** **E709G(4)** **E709H(2)** **E709K(9)** **E709V(2)**	Lung, prostate	JM-kinase linker
**E711K(1)**	Lung	JM-kinase linker
**W731R(1)**	Lung	β2-β3 loop
**P733L(1)** **P733S(1)** **P733T(1)**	Lung	β2-β3 loop
**G735S(3)**	Prostate, Lung	β2-β3 loop
**V738G(2)**	Prostate	β2-β3 loop
**P741H(1)** **P741L(2)**	ThyroidCentral-nervous-system	β3 strandaa
**S768C(1)** **S768I(21)** **S768I(23)** **S768N(1)**	Lung, Oesophagus,Central-nervous-system	C-helix
**S784F(2)** **S784P(1)** **S784Y(1)**	Lung,Upper_aerodigestive_tract	β4-β5 loop
**V802F(2)** **V802I(1)**	Lung,Upper_aerodigestive_tract	αD-helix
**G810D(2)** **G810S(1)**	Upper_aerodigestive_tract,Lung	αE-helix
**P848L(3)** **P848L(4)**	Upper_aerodigestive_tract,Lung	β7-β8 loop
**L861F(1)** **L861P(1)** **L861Q(26)** **L861Q(30)** **L861R(5)** **L861R(3)** **L861V(1)**	Lung,Central-nervous-system	Activation loop
**F968L(1)**	Central-nervous-system	I-helix
**D1012H(1)**	Lung	C-terminal Tail

### Structural analysis of ErbB kinase-conserved residues and proposed roles

**Figure 3:**
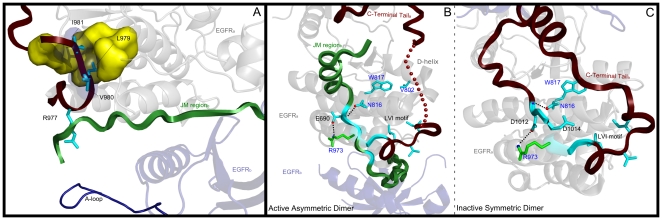
Role of CLT residues in tethering the JM and C-terminal tails. A) Role of the PxR[YF]LVI motif in tethering the C-terminal tail to the C-lobe (PDB:2J5F). B) Tethering of the JM segment to the C-lobe in the active state (PDB: 3GOP). C) Tethering of the C-terminal tail to the C-lobe in the inactive state (PDB: 3GT8). EGFRa and EGFRb correspond to monomer A and monomer B in the asymmetric dimer, respectively. The PxR[YF]LVI motif in the C-terminal tail is labeled as LVI motif. The structure images were generated using Pymol (www.pymol.org). ErbB kinase-specific residues are colored in cyan and hydrogen bonds are depicted as black dotted lines. The disordered segment of the C-terminal tail in the active state (Figure 3B) is shown in dotted representation. Note that R973 is colored in green in Figures 3B and C because this residue is shared by some tyrosine kinases outside of the ErbB family.

To understand how the identified ErbB kinase conserved residues contribute to ErbB kinase functional specialization, and how mutations of these residues contribute to disease, we performed crystal structure analysis of the identified residues (see methods). As shown in Figure 2, nearly all the ErbB conserved residues, although widely dispersed in sequence, spatially interact with the flexible JM and C-terminal tail segments to “tether” them to three regions of the kinase core, namely, the N-lobe, the C-lobe and the active site (Figure 2). Because these interactions are malleable in crystal structures (Table S1), we use the term “tether” [34] to describe these interactions. Broadly, ErbB conserved residues can be classified into three categories based on their structural location and interaction: (i) N-Lobe Tether (NLT): residues that tether the JM and C-terminal tail to the kinase N-lobe; (ii) Active Site Tether (AST): residues that tether the C-terminal tail to the ATP binding site; and (iii) C-Lobe Tether (CLT): residues that tether the JM and C-terminal tail to the kinase C-lobe (Figure 2; Figure 3).

The NLT, AST and CLT residues are highly conserved in ErbB kinases, but strikingly different in RTKs outside of the ErbB family. This selective conservation is likely to be important for ErbB kinase functions, rather than for maintaining ErbB kinase structure or fold, because RTKs that lack these residues essentially adopt the same fold as ErbB kinases [30], [31], [38]. Indeed, recent studies on the activation mechanism of EGFR and related ErbB kinases support the functional importance of some of the identified residues. For example, the C-terminal tail [MF][AC][RK]DPxR[YF]LVI motif, which is part of the CLT, has been shown to play an important role in ErbB3-ErbB2 hetero-dimerization [4], [39], and EGFR activation [11] (Figure 3A). Similarly, D984, a distinctive aspartate (D984) in the C-terminal tail, was recently shown to control C-terminal tail movement and kinase activation [40]. Likewise, N816 and W817 in the CLT have been noted to provide a malleable docking surface for the JM and C-terminal tail in the active [12], and inactive states [11] of EGFR, respectively (Figure 3B-C) [22]. We note that the JM and C-terminal tail docking surface on the C-lobe is coupled to the substrate binding αD-helix [41] via hydrophobic interactions between W817 and V802 in the CLT (Figure 3C).

Whereas the role of CLT residues is well understood, little is known about the role of the NLT and AST residues in ErbB kinase functions. In particular, the selective conservation of residues in the β2-β3 loop and JM-kinase linker are largely mysterious. To obtain insights into these mysterious residues, we performed crystal structure analysis of NLT and AST residues, and interpreted our observations in light of the wealth of functional data available on ErbB kinases. Our analysis suggests important functional roles for the NLT and AST residues, and provides new clues to cancer mutations that alter these residues.

### NLT: A structural framework for coupling C-helix and inter-lobe movement in ErbB kinases

As mentioned earlier, activation of EGFR kinase by dimerization involves conformational changes in key regions of the N-lobe, including repositioning of a regulatory C-helix from an inactive to active conformation, and movement of the N-lobe relative to the C-lobe [11], [42]. We find that these flexible regions of the N-lobe are tethered to the JM and C-terminal tail via ErbB kinase-conserved interactions described below.

#### Interactions tethering the JM and regulatory C-helix

**Figure 4:**
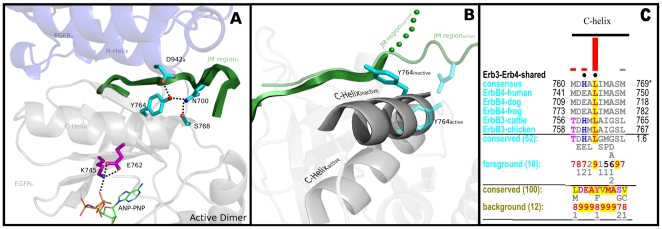
Interactions tethering the JM to the C-helix in the N-lobe. A) Tethering interactions in the active state of EGFR (PDB: 2J5F). The modeled nucleotide (ANP-PNP) is shown in sticks representation. Key hydrogen bonds mediated by ErbB kinase conserved residues are shown. B) Conformational changes associated with ErbB kinase conserved residues in the active (PDB: 2J5F) and inactive state of EGFR (PDB: 2RFE). C) A multiple sequence alignment showing the replacement of Y764 by a leucine in ErbB3 and ErbB4. The foreground alignment in Figure 4C corresponds to ErbB3 and ErbB4 sequences (62 sequences) and the background alignment corresponds to ErbB1 (EGFR) and ErbB2. The NCBI sequence identifiers numbers for the sequences used in the query alignment of Figure 4C are as follows: ErbB4-human: 167745042; ErbB4-dog: 74005688; ErbB4-frog: 147906005; ErbB3-cattle: 156718140; ErbB3-chicken: 113206130.

Tethering of the JM to the C-helix is mediated through ErbB conserved residues in the C-helix and JM segment. In particular, a conserved asparagine (N700) in the JM segment hydrogen bonds to the side-chain of Y764 and S768 in the C-helix (Figure 4A). While these interactions are stable in the active state of EGFR (see Table S1), in the inactive state these interactions are disrupted, in part, because of repositioning of the C-helix in an inactive “out” conformation [11], [42] (Figure 4B). In particular, Y764 in the C-helix moves away from N700 in the inactive state to interact with hydrophobic residues in the β4 strand. This malleable tethering of the JM to the C-helix is likely to be functionally significant, as this may provide a framework for the JM and the activating monomer to dynamically control C-helix movement [11], [12]. Consistent with this view, mutation of Y764 to a phenylalanine [43], or N700 to an alanine [12], have been found to significantly impair EGFR kinase activity. Notably, ErbB3 and ErbB4 conserve a leucine at the Y764 position (Figure 4C). This variation may reflect the unique ability of ErbB3 and ErbB4 to form inactive N-lobe-N-lobe dimers, as the leucine, which replaces Y764, is part of the N-lobe-N-lobe dimer interface in ErbB3 and ErbB4 [15], [16], [17].

#### Oncogenic mutations, S768I and L861Q, may alter the canonical interactions at the JM-C-helix interface

*S768I*: S768I is a frequently occurring mutation (Table 1) in the C-helix of EGFR that increases basal kinase activity [44]. S768 is located at the asymmetric dimer interface and is known to get phosphorylated by Calcium calmodulin-dependent kinase II (CAMK2), which modulates EGFR autokinase activity by phosphorylation of S768 and C-terminal tail serine residues [45]. Thus, mutation of S768 to isoleucine can contribute to abnormal EGFR kinase activity by impacting one or more of the following functions: (i) altering the tethering interactions between the JM and C-helix, (ii) changing the dimer interface [46], and (iii) preventing CAMK2 phosphorylation.

**Figure 5:**
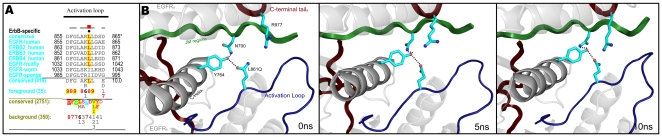
Selective conservation and modeling of L861Q mutation (based upon PDB: 2JIU and 2ITN). A) Selective conservation of L861 in the ErbB family. B) Local structural interactions mediated by the L861Q lung cancer mutation in EGFR and snapshots of this interaction at various time points (0, 5 and 10 ns) during the MD run. RMSD plots of the backbone atoms during the course of the simulation for the active (dimer) and inactive (monomer) are shown in Figure S1.

*L861Q*: L861Q is a frequently occurring activating mutation in the activation loop of EGFR [47]. L861 is specific to ErbB kinases (Figure 5A) and is typically conserved as an aspartate (D) in RTKs outside of the ErbB family (Figure 5A). In the inactive state of EGFR, L861 packs up against hydrophobic residues in the C-helix [20], and this observation previously led to the suggestion that the L861Q mutation may activate EGFR by destabilizing the hydrophobic interactions in the inactive state [10], [46], [48]. However, the structural interactions that stabilize the active form of the L861Q mutant have not been proposed before. Modeling of a glutamine in the active form of EGFR indicates that a glutamine at the L861 position can potentially form a hydrogen bond with Y764 (in the C-helix) in the active form, but not in the inactive form of EGFR (Figure 5B). Furthermore, molecular dynamics studies on the L861Q mutant (Figure S1) indicates that the hydrogen bond between Q861 and Y764 is stable during the course of the simulation (Figure 5C), and can likely prevent Y764 from switching to an inactive conformation (Figure 5B-D). Thus, in addition to destabilizing the inactive state, the L861Q mutation may activate EGFR by stabilizing the C-helix tyrosine (Y764) in an active conformation. We also predict that the L861Q mutation in ErbB3 and ErbB4 may not have the same functional impact as in EGFR because ErbB3 and ErbB4 conserve a leucine at the Y764 position (Figure 4C).

#### Interactions tethering the C-terminal tail and inter-lobe hinge

The opening and closing of the N-terminal ATP binding lobe relative to the C-terminal substrate-binding lobe is an essential part of catalysis [23]. Inter-lobe movement in eukaryotic protein kinases is facilitated by the inter-lobe linker [49], which connects the N and C lobes, and lobe-spanning salt bridges, which serve as hinge points for domain movements [50].

**Figure 6:**
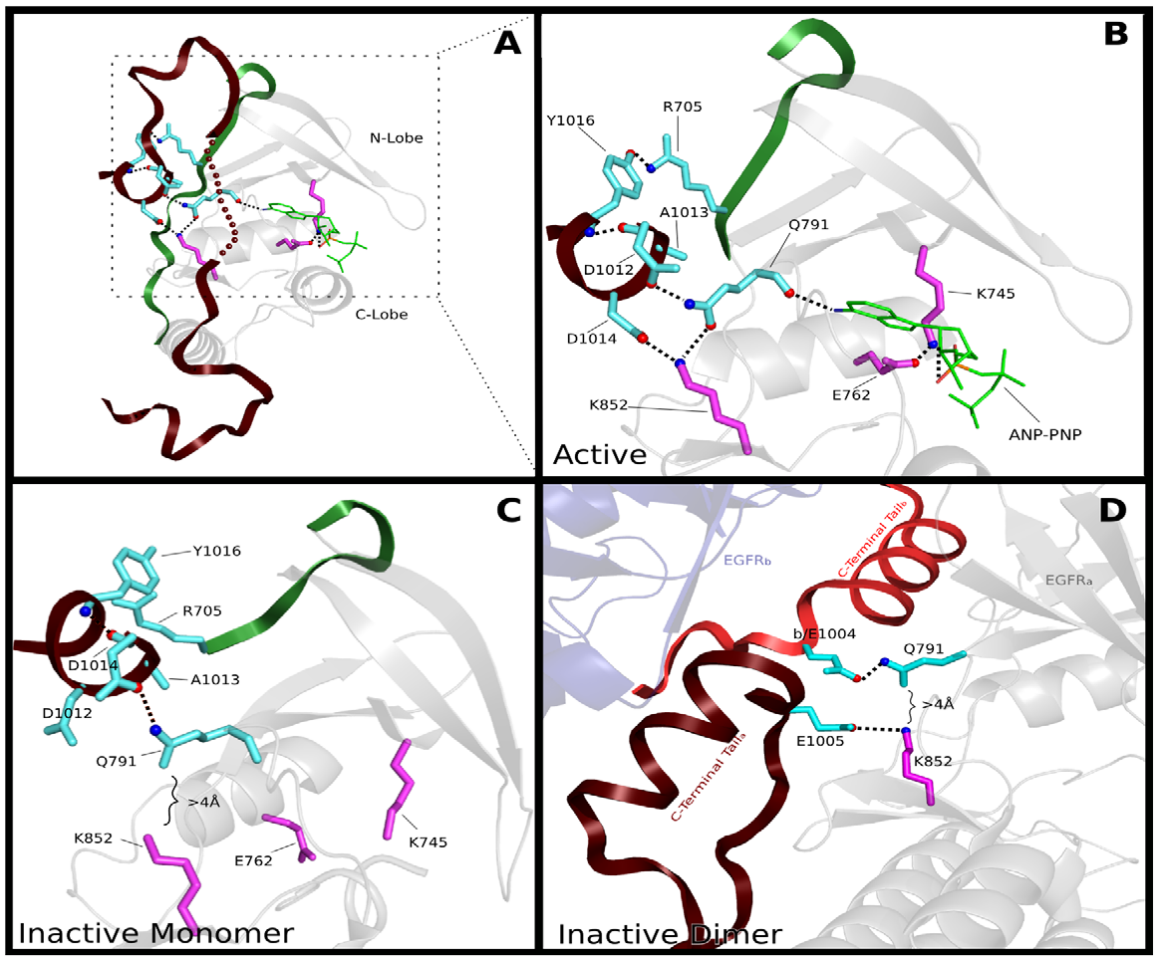
Interactions tethering the C-terminal tail to hinge regions of the kinase core. A) Structural location of the hinge tether. B-D) A close-up view of the tethering interactions in the (B) active dimer (PDB: 2ITN), (C) inactive monomer (PDB: 2RFD), and (D) inactive dimer (PDB: 3GT8). ANP-PNP is shown as green sticks and hydrogen bonds are depicted as black dotted lines. Some hydrogen bonds have been omitted for clarity.

In ErbB kinases, the hinge regions of the kinase domain are tethered to the C-terminal tail via ErbB kinase-conserved residues (Figure 6A). In particular, an ErbB kinase-conserved glutamine (Q791) tethers the C-terminal tail to the inter-lobe linker by hydrogen bonding to two conserved asparates (D1012 and D1014) in the C-terminal tail (Figure 6B). In EGFR, one of the aspartates (D1014) in the C-terminal tail also hydrogen bonds to a kinase conserved lysine (K852 in the C-lobe), which has been noted to serve as a pivot point for inter-lobe movement [50]. Thus, ErbB kinases have diverged from other RTKs to uniquely tether the C-terminal tail to hinge regions of the kinase domain critical for inter-lobe movement. Why would such tethering be important for ErbB functions? One possibility is that this may provide an additional layer of regulation by allowing the C-terminal tail to internally control inter-lobe movement, and consequently kinase activity. Notably, in the inactive structure of EGFR, where the two lobes are in a closed conformation, the lobe-spanning salt bridge between the glutamine (Q791) and the lysine (K852) is lost, in part because of the movement of C-terminal tail away from the lysine (K852) (Figure 6C) [42]. Also, the C-terminal tails in the inactive dimer [11] prevent the formation of inter-lobe salt bridge by engaging Q791 and K852 in different interactions (Figure 6D).

The control of inter-lobe movement by the C-terminal tail is likely to differ in parasitic worms and sponges because the C-terminal tail residues that interact with the inter-lobe hinge are different in these organisms. Sponges lack the C-terminal tail aspartates (D1012 and D1014), and parasitic worms contain a glutamine at the D1014 position. Notably, both sponges and parasitic worms replace the glutamine (Q791) in the inter-lobe linker by an isoleucine (I) and glutamate (E), respectively (Figure 1). Although the functional implication of this lineage specific variation is unclear, it is likely that EGFR orthologs in sponges and parasitic worms do not require regulation of catalytic activity by the C-terminal tail. We note that ErbB3 differs from other ErbB members in the inter-lobe hinge. In particular, the kinase conserved lysine (K852), which forms a lobe spanning salt bridge with Q791, is conserved as a glutamine (Q) in ErbB3. This ErbB3-specific variation may contribute to the low levels of kinase activity [17] by preventing opening and closing motion during catalysis.

#### Interactions coupling the C-terminal tail and the JM segment

**Figure 7:**
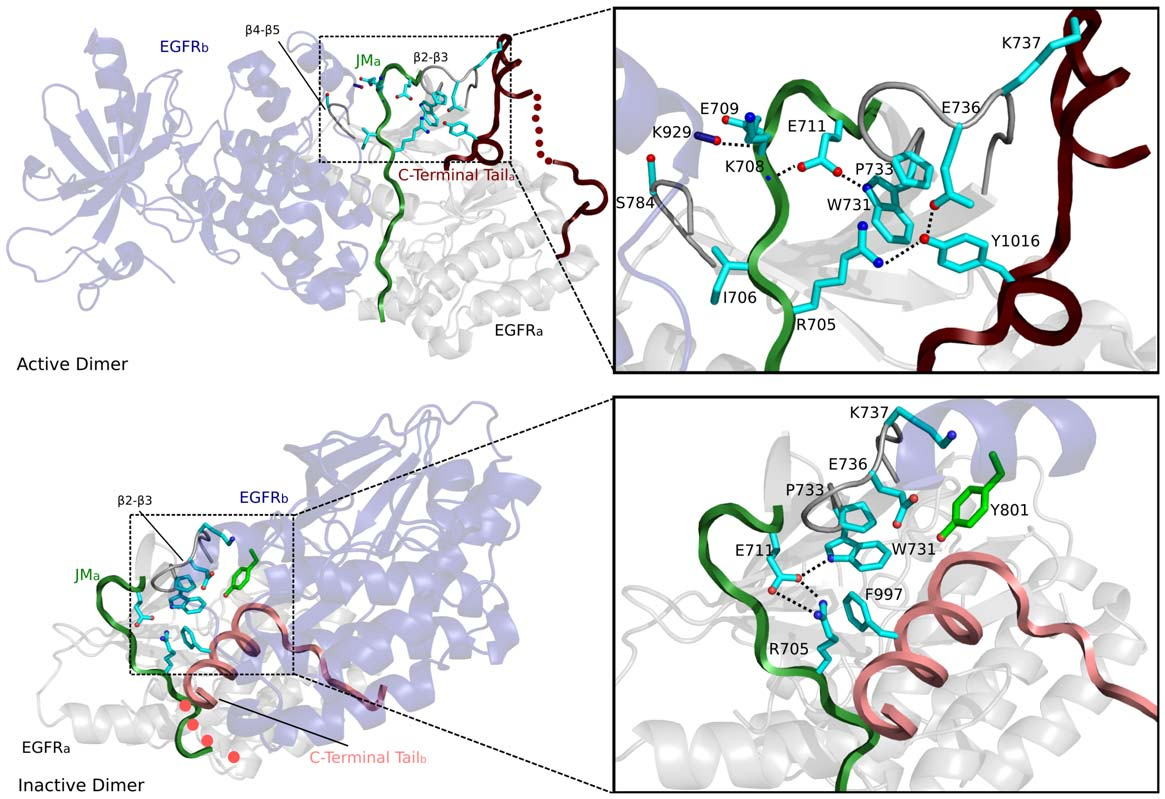
Interactions tethering the C-terminal tail, JM-kinase linker and the β2-β3 loop (labeled as beta2-beta3). The top panel shows structural interactions associated with ErbB kinase-conserved residues in the active asymmetric dimer (PDB: 2ITN) and the bottom panel shows the inactive dimer (PDB: 3GT8). The right panel shows a close-up view of the interactions.

As mentioned earlier, some of the strongest ErbB kinase-specific constraints are imposed on residues in the JM-kinase linker and β2-β3 loop. ErbB-conserved residues in these two regions structurally couple a phosphorylatable tyrosine (Y1016) in the C-terminal tail to the JM and N-lobe regions involved in dimerization (Figure 7). Some of the distinctive residues involved in this coupling include, R705 in the JM-kinase linker, W731 in the β2-β3 loop, and E736 in the β2-β3 loop. Specifically, R705 and E736 form hydrogen bonds with the hydroxyl group of Y1016, and W731 provides a favorable docking surface for the aromatic ring of Y1016. These interactions are further coupled to the C-helix and dimerization sites in the N-lobe by I706 and E711 in the JM-kinase linker (Figure 7). Specifically, I706 packs up against hydrophobic residues in the C-helix, and E711 hydrogen bonds to the side-chain of W731, as well as to the backbone of K708, which is involved in the asymmetric dimer interface [10], [12] (Figure 7).

The canonical ErbB kinase-conserved interactions between the C-terminal tail, JM-kinase linker, and β2-β3 loop are altered in the inactive dimer [11] (Figure 7). In particular, a conserved C-terminal tail phenylalanine (F997) occupies a position analogous to Y1016 in the inactive state (Figure 7). ErbB kinase-conserved residues in JM-kinase linker and β2-β3 loop also undergo concerted changes in the inactive dimer. In particular, R705, which hydrogen bonds to Y1016 in the active state, moves away to interact with E711 in the JM-kinase linker. Likewise, E736, which typically interacts with Y1016 in the active state, moves towards a phosphorylatable tyrosine (Y801 in the D-helix) in the inactive state [11]. Together, these concerted changes appear to dynamically couple the C-terminal tail with the JM and C-helix regions involved in dimerization. Notably, a similar coupling between the SH2-kinase linker, β2-β3 loop and SH3 domain have been noted for Src tyrosine kinase [51], [52], where the SH3 domain performs a function analogous to the C-terminal tail of EGFR [11].

Our analysis suggests that the structural coupling between the C-terminal tail and JM-kinase linker (described above) is likely to differ in ErbB3, and EGFR orthologs in sponges and parasitic worms because ErbB3 conserves an aspartate at the Y1016 position, and EGFR orthologs in sponges and parasitic worms lack some of the canonical ErbB residues in the JM-kinase linker and β2-β3 loop (Figure 1). Our analysis also suggests that cancer mutations in the β2-β3 loop and the JM-kinase linker (Table 1) may contribute to abnormal regulation by altering the conformational coupling between these two regions.

### AST: A hypothetical mechanism for regulating ATP binding by the C-terminal tail

**Figure 8:**
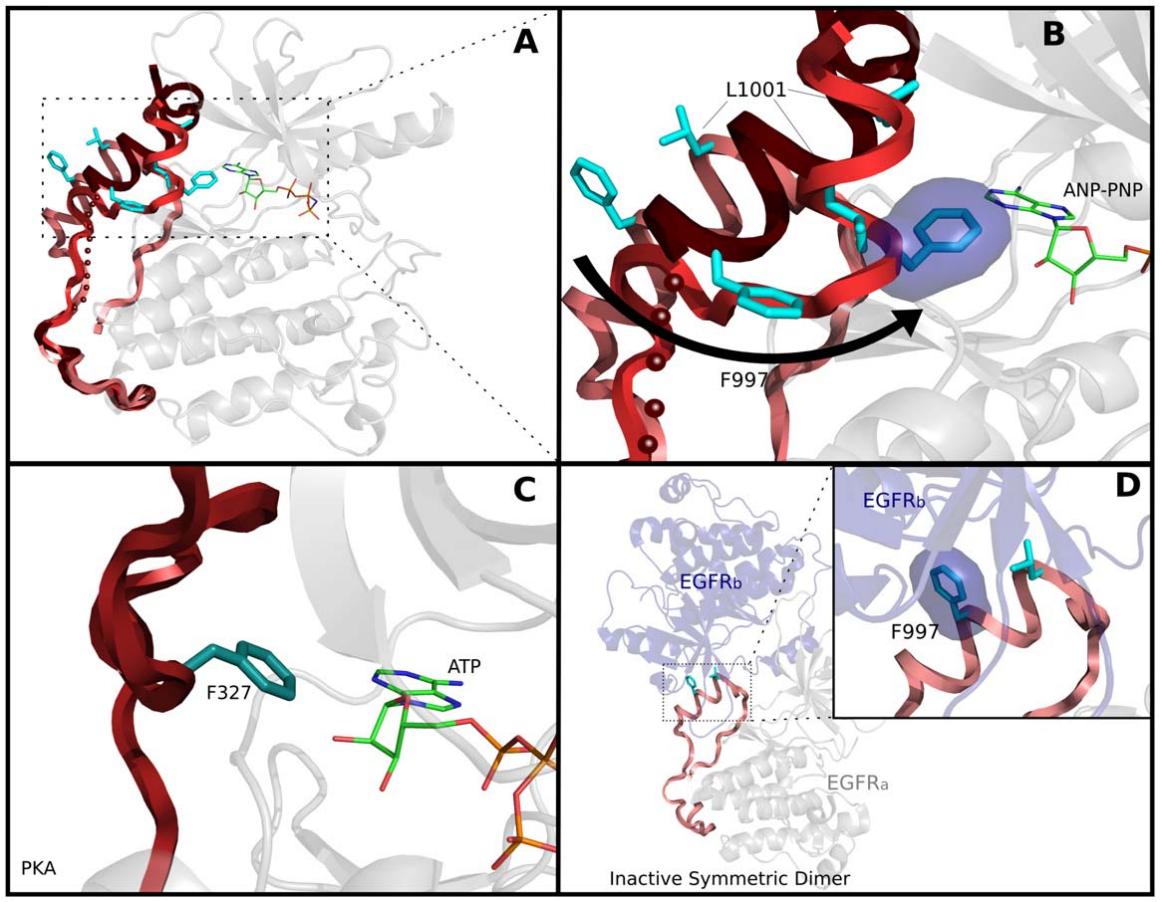
Active site tether (AST) and its role in ATP binding. A) Superposition of the C-terminal tail in the active (PDB: 2JIU), inactive monomer (PDB: 1XKK) and inactive dimer (PDB: 3GT8). B) A close up view of F997 and its proposed role in ATP binding. The modeled ANP-PNP is shown in sticks representation. C) Structural location of F327 (PKA numbering) in the C-terminal tail of PKA (PDB: 1ATP [49]). D) Conformational changes associated with F997 in the inactive symmetric dimer (PDB: 3GT8).

The AST is largely formed by a helical segment (residues 997–1001) in the C-terminal tail (Figure 8A), also referred to as the AP-2 helix [11]. The AST is typically disordered in most ErbB structures; however, in two structures of EGFR (PDB:1XKK and 2JIU) [19], [53], the AST segment adopts two distinct conformations. In one conformation, it protrudes into the ATP binding pocket, thereby tethering the C-terminal tail to the ATP binding site (via two hydrophobic residues, F/L997 and L1001), while in the other conformation the AST swings away from the ATP binding pocket to become solvent-exposed (Figure 8A–B) [19], [53]. This mode of dynamically tethering the C-terminal tail to the ATP binding pocket is remarkably similar to PKA (Figure 8C), where a conserved phenylalanine (F327^PKA^) in the C-terminal tail moves in and out of the ATP binding pocket to serve as a gate for nucleotide binding [23], [54]. An analogous role for F997 in EGFR would suggest a similar gating mechanism, wherein nucleotide binding is controlled in a conformation dependent manner by the AST. Such a function may also explain the paradoxical experimental observations, where mutations in the AST both increases [11] and decreases catalytic activity [10], [55].

### Concluding Remarks

**Figure 9:**
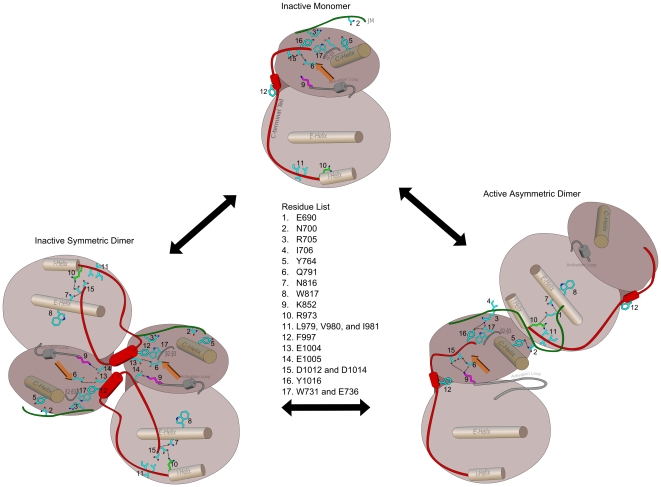
Mechanistic model of EGFR kinase activation involving ErbB kinase-conserved residues. Three functional states of EGFR are used (active dimer based upon PDB: 3GOP), inactive dimer (based upon PDB: 3GT8) and inactive monomer (based upon PDB: 1XKK) to illustrate the coordinated functions of the identified residues in kinase activation. The JM and C-terminal tails are colored in green and red, respectively. The residue numbers for the labeled residues are shown. The opening and closing motion of the two lobes are shown to illustrate how the C-tail may regulate catalytic activity in a conformation dependent manner.

Bayesian analysis of the evolutionary constraints acting on receptor tyrosine kinase sequences has revealed a co-conserved pattern characteristic of the ErbB family. Analysis of this co-conserved pattern, in light of the wealth of structural and functional data available on ErbB kinases, suggests a model in which the identified residues contribute to ErbB kinase functional divergence by providing a structural framework for the JM and C-terminal tail to uniquely regulate ErbB kinase activity (Figure 9). A compelling aspect of this model is that it readily explains the inhibitory and activating functions of the C-terminal tail segment, and provides new testable hypotheses for experimental studies. For example, the hypothesis that the ErbB kinase-conserved residues contribute to the *cis* regulation of the kinase core by the JM and C-terminal tail can be tested experimentally. Likewise, the hypothesis that the activation mechanism of sponges and roundworms differs from their mammalian counterparts can also be tested experimentally. Finally, by identifying a potential role for the C-terminal tail in ATP binding (AST), our study provides new avenues for designing selective ErbB kinase inhibitors.

## Materials and Methods

### Identification of ErbB-specific selective constraints

ErbB and related receptor tyrosine kinase (RTK) sequences from diverse organisms were identified within NCBI nr, env_nr, and translated EST databases using PSI-BLAST and motif-based search procedures. These sequences were multiply aligned using the MAPGAPS program [56] by building curated alignments/profiles for each of the 18 families within the RTK sub-group. These alignments included the Juxtamembrane region, the kinase domain and ∼50 residue segments C-terminal to the kinase domain. The aligned sequences (∼3,170 sequences) were partitioned into two functionally divergent subgroups using a Bayesian partitioning with pattern selection (BPPS) procedure [57]. This identified a distinctive pattern that most optimally distinguishes ErbB kinase sequences from other receptor tyrosine kinase sequences (Figure 1). The extent to which these residues contribute to the divergence of ErbB kinases was quantified using a ball-in-urn statistical model [58], and indicated by the height of the histogram above the alignments in Figures 1, 4C, and 8A.

### Visualization of ErbB-specific selective constraints

The residues that contribute the most to ErbB kinase evolutionary divergence are shown using a “Contrast Hierarchical Alignment (CHA)” [58] (Figure 1). A CHA is based on three categories of related sequences: (i) a query set, (ii) a foreground set and (iii) a background set. In the Figure 1 alignment, representative ErbB sequences from diverse organisms constitute the query set, all ErbB kinase sequences (419 sequences) correspond to the foreground set, and receptor tyrosine kinases outside of the ErbB family (2,751 sequences) correspond to the background set. The residues that contribute to ErbB kinase evolutionary divergence, as identified by the BPPS procedure, are shown by block dots above the alignment. Notably, the residues identified by the BPPS procedure are highly conserved in the ErbB family (foreground alignment) and strikingly different in receptor tyrosine kinases outside of the ErbB family (background alignment) (Figure 1).

ErbB3 is considered an atypical member of the ErbB family. To determine to what extent ErbB3 contributes to the divergence of the ErbB family, we ran the BPPS procedure by removing ErbB3 sequences from our alignments. Removing ErbB3 sequences did not significantly alter the pattern-partitions created by the BPPS procedure.

### Identification of ErbB3-ErbB4 shared patterns

Representative sequences from ErbB3 and ErbB4 (query set) were multiply aligned against mammalian ErbB kinase sequences (∼164 sequences). The BPPS procedure was applied on this alignment to identify sequence patterns that most distinguish ErbB3 and ErbB4 from other ErbB members (ErbB1 and ErbB4). Among other residues, L794 in the C-helix (Figure 4C) was identified as one of the most contributing residues to ErbB3-ErbB4 functional divergence.

### Structural analysis of ErbB kinase conserved residues

Crystal structures of ErbB kinases solved in various function states (see below) were obtained from the PDB database (http://www.rcsb.org). Protein hydrogen bonds were added to structural coordinates using the Reduce program [59]. Hydrogen bonds, van der Waals interaction and CH-π interaction were calculated using the CHAIN suite of programs [58]. The identified interactions were further quantified by calculating the frequency of occurrence of each interaction across multiple crystal structures (Table S1). The structural interactions were visualized using PyMOL (http://www.pymol.org). The symmetry related molecules in Figs 3b, 4a, 5b and 7a were generated using the “symmetry mates” utility in PyMOL. The following PDB files were used in our analysis:

Active State: 1M14 [4]; 1M17 [4]; 2EB2(not published); 2EB3(not published); 2GS2 [10]; 2GS6 [10]; 2ITN [60]; 2ITO [60]; 2ITP [60]; 2ITQ [60]; 2ITT [60]; 2ITU [60]; 2ITV [60]; 2ITW [60]; 2ITX [60]; 2ITY [60]; 2ITZ [60]; 2J5E [61]; 2J5F [61]; 2J6M [60]; 2JIT [53]; 2JIU [53]; 3GOP [12]

Inactive State: 1XKK [18]; 2GS7 [10]; 2JIV [53]; 2RF9 [42]; 2RFD [42]; 2RFE [42]; 2RGP [62]; 3BEL [63]; 3GT8 [11]

### Retrieval of cancer mutations, modeling and molecular dynamics simulations of the mutant forms

Cancer-associated mutations in EGFR were identified by mining the COSMIC database [64], a repository for somatic mutations in human cancers. A structural model of the lung cancer-associated L861Q mutant was built using two crystal structures of active EGFR kinase domain (PDB ID: 2ITN and 2JIU) as templates. Because all available crystal structures of EGFR have disordered regions, we chose structures with non-overlapping disordered regions (PDB: 2ITN and 2JIU) for our modeling studies. ANP-PNP was modeled in some of the nucleotide unbound structures to show the proximity of ErbB conserved residues to the ATP binding pocket. ANP-PNP was modeled by superimposing the nucleotide unbound structure to the ANP-PNP bound structure of EGFR (PDB: 2ITN). Superposition was done using the “cealign” plugin in Pymol.

For molecular dynamics studies, water molecules, bound inhibitors, and other heteroatom's were removed. The missing residues were modeled using MODELLER [65]. Missing hydrogen and heavy atoms were added using the LEaP program in the Amber software suite [66]. Each protein was solvated with TIP3P water model [67] and counterions were added for neutralization. Molecular dynamics (MD) simulations were done using NAMD software [68], version 2.7b1, and all-atom ff03 force fields from the Amber package. Prior to the regular MD production run, a smoothing function was applied to both the electrostatic and the van der Waals forces at a distance of 10 Å, and a pair list distance of 14 Å with a switching cutoff distance of 12 Å. All bonds with hydrogen were kept rigid by applying the ShakeH algorithm, and the protein backbone atoms were restrained with a harmonic restraint (k_f_ = 10 kcal/(mol · Å^2^)). Conjugate-gradient energy minimization was performed on the solvated protein for 10,000 steps, followed by heating from 0 to 298.15 K. The restraints on the protein backbone atoms over multiple stages of equilibration under NPT ensemble (P = 1 atm, T = 298.15 K) were released to obtain a relaxed protein. The unrestrained MD productions were run for 10 ns using a time step of 2 fs and the NPT ensemble. Root-mean-square deviation (RMSD) calculations were performed and monitored to ensure that the simulation was stable during the 10ns time scale. Hydrogen bonding analysis was done using the ptaj program in Amber suite of programs.

## Supporting Information

Table S1 (0.09 MB DOC)

Figure S1 (0.07 MB DOC)
