# Supplementary material for: Co-Conserved Features Associated with cis Regulation of ErbB Tyrosine Kinases
Source: PLoS One. 2010 Dec 13;5(12):e14310. doi: 10.1371/journal.pone.0014310 (PMC3001462; doi:10.1371/journal.pone.0014310)
Supplement: Table S1 — (0.09 MB DOC) [file pone.0014310.s001.doc]

Table S1: Quantification of hydrogen bonding interactions observed in crystal structures

| **Region** | **Hydrogen Bond** | **Occupancy (Active)**  *% (found/total)* | **Occupancy (Inactive)**  *% (found/total)* | **Found in PDB**  *Active/Inactive* |
| --- | --- | --- | --- | --- |
| Hinge | D1012 – Q791 | 90.48% (19/21) | 11.11% (1/9) | All except 2JIU(B), 1M14(A), 1M17(A), 2JIT(A,B) / 2RFD(A) |
|  | D1014 – Q791 | 94.74% (18/19) | 85.71% (6/7) | All except 1M14(A), 1M17(A), 2JIU(A,B), 2JIT(A,B) / 2RGP(A), 2RFE(D), 3BEL(A), 1XKK(A), 2GS7(B), 2RFD(B) |
|  | D1014 – K852 | 89.74% (17/19) | 0% (0/9) | All except 2JIU(A,B), 1M14(A), 1M17(A), 2JIT(A,B), 2J5E(A) / none |
|  | Q791 – K852 | 70.83% (17/24) | 0% (0/19) | 2ITP(A), 1M14(A), 2ITX(A), 2GS2(A), 2EB2(A), 2ITW(A), 2GS6(A), 2EB3(A), 2JIU(B), 2ITN(A), 2J6M(A), 2ITT(A), 2ITV(A), 2ITU(A), 2JIT(B), 2ITQ(A), 1M17(A) / none |
| β2-β3 Loop | E711 – W731 | 100% (22/22) | 89.47% (17/19) | All except 2JIU(B), 2JIT(B) / All except 2RGP(A), 2RFE(C) |
|  | E711 – K708 | 100% (22/22) | 94.74% (18/19) | All except 2JIU(B), 2JIT(B) / All except 2JIV(B) |
|  | E711 – R705 | 0% (0/22) | 21% (4/19) | None / 3GT8(A,B,C,D) |
|  | R705 – Y1016 | 38.1% (8/21) | 33.3% (2/6) | 2ITP(A), 2ITX(A), 2GS2(A), 2EB3(A), 2ITN(A), 2J6M(A), 2ITT(A), 2ITV(A) / 2GS7(B), 2RFD(B) |
|  | E736 – Y1016 | 94.74% (18/19) | 0% | All except 2JIU(A,B), 1M14(A), 1M17(A), 2JIT(A,B) / none |
| C-Helix | S768 – N700 | 100% (20/20) | 0% | All except 2JIU(A,B), 2JIT(A,B) / none |
|  | Y764 – N700 | 65% (13/20) | 0% | 1M14(A), 2ITX(A), 2GS2(A), 2EB2(A), 2EB3(A), 2ITO(A), 2J5F(A), 2J6M(A), 2J5E(A), 2ITT(A), 2ITV(A), 2ITZ(A), 1M17(A) / none |

Note that only crystallographically resolved residues were considered for occupancy calculations.

***Amar et.al.* Suppl. Figure 1**

Suppl. Figure 1: RMSD profile versus time for the protein backbone atoms in the active (dimer) and inactive (monomeric) forms of the L861Q mutant.
