# Supplementary material for: Co-Conserved Features Associated with cis Regulation of ErbB Tyrosine Kinases
Source: PLoS One. 2010 Dec 13;5(12):e14310. doi: 10.1371/journal.pone.0014310 (PMC3001462; doi:10.1371/journal.pone.0014310)
Supplement: Figure S1 — (0.07 MB DOC) [file pone.0014310.s002.doc]

**Figure S1**

Figure S1: RMSD profile versus time for the protein backbone atoms in the active (dimer) and inactive (monomeric) forms of the L861Q mutant simulations.
